# ArmourTraits: A comparative dataset on the ecological and evolutionary correlates of dermal armour in squamates

**DOI:** 10.1016/j.dib.2026.112904

**Published:** 2026-05-30

**Authors:** Chris Broeckhoven, Bryan Minne, Cang Hui

**Affiliations:** a BiorteX, Swellendam 6740, South Africa; b Department of Botany and Zoology, Stellenbosch University, Stellenbosch 7602, South Africa; c Herpetological Education and Research Project (H.E.R.P.) BV, Sint-Gillis-Waas 9170, Belgium; d Centre for Invasion Biology, Department of Mathematical Sciences, Stellenbosch University, Stellenbosch 7602, South Africa; e Biodiversity Informatics Unit, African Institute for Mathematical Sciences, Cape Town 7945, South Africa; f National Institute for Theoretical and Computational Sciences, Stellenbosch 7602, South Africa

**Keywords:** Squamata, Comparative morphology, Evolution, Functional morphology, Predator-prey interactions

## Abstract

The evolution of defensive traits is a central topic in evolutionary biology, yet quantitative data linking variation in defensive morphology to ecological and environmental factors remain limited. *ArmourTraits* is a comprehensive dataset that quantifies variation in body armour across 131 species from two distantly related squamate lineages, Cordyliformes and Anguimorpha, which convergently evolved armour in the form of osteoderms. The dataset integrates morphological measurements of osteoderm expression and hindlimb skeletal traits derived from micro-computed tomography scans, species-level ecological, life-history, and environmental data, as well as estimated predation risk and a time-calibrated phylogeny. By providing standardised, quantitative metrics of defensive morphology alongside locomotor traits and associated ecological variables, *ArmourTraits* enables phylogenetic comparative analyses of ecological correlates, functional trade-offs, convergent evolution, and the diversification of defensive traits across squamates.

Specifications Table

**Table:** 

Subject	Biology
Specific subject area	Ecology and evolutionary biology
Type of data	Table, Excel Raw
Data collection	Morphological data, including osteoderm and hindlimb traits were obtained from 272 specimens using high-resolution micro-computed tomography. Environmental variables were extracted from WorldClim, MODIS, and CGIAR-CSI databases in R. Predation risk was calculated using spatial overlap and trait-based filtering of predator–prey pairs. Life-history and phylogenetic data were compiled from published trait datasets and a time-calibrated squamate phylogeny.
Data source location	Institution: Stellenbosch University City/Town/Region: Stellenbosch Country: South Africa
Data accessibility	Repository name: Zenodo Data identification number: 10.5281/zenodo.20407495 Direct URL to data: https://zenodo.org/records/20407495
Related research article	None

## 1 Value of the Data

• This dataset provides quantitative measurements of defensive traits across two major armoured squamate lineages, Cordyliformes and Anguimorpha, spanning 131 species. Using micro-computed tomography (micro-CT), the dataset quantifies the expression of osteoderms or dermal armour, together with body size and hind limb measurements. These data provide a detailed morphological resource for studying variation in defensive morphology.
• The dataset integrates morphological data with environmental variables, estimated predation risks, and life history traits, enabling researchers to investigate how defensive morphology is shaped by climate, predation, habitat, and other ecological factors. These data can support ecological and evolutionary analyses examining the environmental correlates of defensive traits across two lineages of squamates.
• By spanning two distantly related squamate clades that independently evolved dermal armour, the dataset provides a valuable resource for phylogenetic comparative studies of convergent evolution and the diversification of defensive traits.
• Detailed measurements of osteoderm expression across functionally important body regions, combined with locomotor apparatus data, allow researchers to explore functional trade-offs between defence and locomotor performance. This enables testing of hypotheses about how factors such as predation risk and/or climatic conditions may favour investment in armour versus speed.

## 2 Background

The evolution of animal armour has long fascinated scientists, yet little is known about the ecological and evolutionary drivers that shape defensive morphologies [1]. It remains puzzling why some species—or even entire lineages—evolved armour while others did not. Traditionally, armour has been viewed as an adaptation for protection against predators; however, recent comparative studies in squamates suggest that defensive morphologies do not necessarily evolve in response to direct predator-prey interactions [2]. Habitat-mediated predation risk, in which habitat use alters vulnerability to predators, can influence armour expression [2], and other physiological factors unrelated to predation—such as thermoregulation, water retention, or mineral storage—may also play important roles [1,3]. By considering the ecological and evolutionary context, along with the functional consequences of armour, researchers can better understand the complex interplay of selective pressures driving the diversification of defensive traits. Here, we present a unique dataset spanning two highly variable squamate clades that independently evolved dermal armour in the form of osteoderms, bony elements embedded in the skin, providing a model system to investigate the evolution and functional trade-offs of defensive morphologies.

## 3 Data Description

The main dataset (Excel workbook) comprises several sheets.

**Morphology** provides the raw morphological measurements for all scanned specimens together with associated taxonomic information. Measurements include osteoderm metrics, hindlimb dimensions, and estimates of body size. Specimens in which the longest hind toe was missing are indicated with ‘n/a’.

**Scan Settings** summarises metadata for each specimen, including collection number (where available), spatial resolution of the micro-CT scan, scanning system and facility, and the source of the scan (i.e., MorphoSource, newly generated for this study, or obtained from previous studies).

**Life-history** contains categorical trait data for each species, including activity pattern (diurnal, cathemeral, or nocturnal), habitat use (semifossorial, terrestrial, saxicolous, semiarboreal, or arboreal), foraging strategy (sit-and-wait or active), reproductive mode (oviparous or viviparous), sociality, island endemism, and the presence or absence of a serpentiform body plan.

**Climate** provides environmental variables extracted for each species, including temperature-related variables, precipitation-related variables, and additional environmental descriptors including elevation, average annual solar radiation, average annual wind speed, cloud cover, global aridity index, and percentage tree cover.

**Predation Risk** contains estimated predation risk metrics for each species, including predation risk scores for birds, mammals, and snakes, total predation risk, and the relative contribution of each predator guild (birds, mammals, snakes) to overall predation risk.

In addition, three separate Excel workbooks provide the intermediate calculations used to derive predation risk indices for each predator guild. These comprise three sheets: Predation Risk – Birds, Predation Risk – Mammals, and Predation Risk – Snakes, each containing predator–prey interaction scores.

A phylogenetic tree file (.tree) is provided as a separate file and contains the time-calibrated phylogeny of the taxa used in the main dataset, with and without outgroups included.

## 4 Experimental Design, Materials and Methods

### 4.1 Morphological data

Micro-computed tomography (micro-CT) scans were obtained for 272 specimens representing Cordyliformes (*n* = 68 species; 64% of total diversity) and Anguimorpha (*n* = 63 species; 25% of total diversity). Specimens were obtained from multiple institutional collections, including the Ellerman Collection at Stellenbosch University, the Port Elizabeth Museum (Bayworld), the Royal Belgian Institute of Natural Sciences, the Museum Koenig Bonn, the American Museum of Natural History and the Field Museum of Natural History, as well as from private collections of the authors (CB and BM). These specimens were either scanned as part of the present study or obtained from previously published studies by the author(s) [2]. To increase taxonomic coverage, additional micro-CT datasets were obtained from the online repository MorphoSource. Scans were acquired using high-resolution micro-CT scanners at participating institutions (see accompanying dataset). Reconstructions were performed using scanner-specific software. Micro-CT datasets were subsequently processed and analysed in VGStudio MAX 3 (Volume Graphics GmbH, Heidelberg, Germany) to quantify defensive morphology (osteoderm expression) and locomotor morphology (hindlimb skeletal dimensions).

**Fig. 1:**
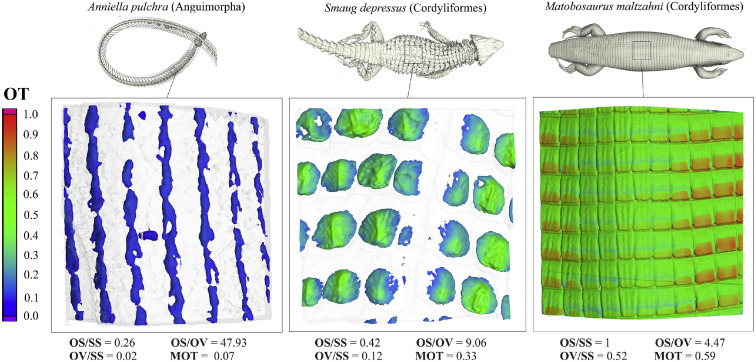
Representative micro-CT visualisations illustrating the range of osteoderm expression observed across the dataset. The top row shows whole-body 3D reconstructions with the sampled region of interest (ROI) on the dorsal trunk indicated. The bottom row shows magnified views of the extracted skin ROI (transparent) with segmented osteoderms colour-coded by osteoderm thickness (OT, mm). For each ROI, the following osteoderm expression metrics are reported: osteoderm surface area relative to skin surface (OS/SS), osteoderm volume relative to skin surface (OV/SS), osteoderm surface area relative to osteoderm volume (OS/OV), and mean osteoderm thickness (MOT). These examples illustrate the morphological variation captured by the quantitative parameters used in this study.

For osteoderm expression, four region-of-interest (ROI) areas of skin were digitally extracted per specimen (Fig. 1): two from the neck (dorsal and ventral surfaces) and two from the trunk (dorsal and ventral surfaces). The tail region was excluded because armour in this region is strongly associated with microhabitat use in squamates [4], and therefore represents a functionally distinct axis of variation [2]. Unlike previous approaches using fixed-size ROIs [2], ROI dimensions were relative to specimen size to account for interspecific variation in body size. Each ROI measured approximately half the width of the corresponding body region to capture a representative sample of osteoderms in that region. ROIs were positioned centrally within each region to avoid transitional zones near the head, limbs, or tail base, and were placed on relatively flat skin areas to minimise curvature effects. Mineralised tissue boundaries were identified via surface determination, and osteoderms were segmented manually with thresholds adjusted individually for each scan. Where necessary, noise was removed using the Polyline 3D function implemented in VGStudio MAX. Four morphometrics describing osteoderm expression were quantified for each ROI: osteoderm surface area relative to skin surface (OS/SS), osteoderm surface area relative to osteoderm volume (OS/OV), osteoderm volume relative to skin surface (OV/SS) and osteoderm thickness (OT). Osteoderm volume (OV) was extracted directly from the segmented osteoderm structures in VGStudio MAX. Skin surface area (SS) corresponded to the area of skin tissue encompassed by the ROI and was defined as the projected area of the ROI onto an orthogonal dorsal plane. To estimate osteoderm surface coverage (OS/SS), dorsal-view images of the segmented osteoderms were exported from VGStudio MAX and analysed in ImageJ 1.54f (National Institutes of Health, USA). Images were cropped to the ROI boundaries and converted to binary format to separate osteoderm material from the background. Thresholding was applied to isolate osteoderm pixels, after which the proportion of the ROI occupied by osteoderms (OS/SS) was calculated as the number of osteoderm pixels divided by the total number of pixels within the ROI. Mean osteoderm thickness (MOT) was estimated using the wall-thickness analysis implemented in VGStudio MAX (sphere method), which calculates local thickness as the diameter of the largest sphere that can be inscribed within the structure. The mean value of the resulting thickness distribution was used as the representative osteoderm thickness for each ROI.

For hindlimb skeletal morphology, several elements were measured from the micro-CT scans, including ilium length, femur length, tibia length, metatarsus length, and the length of the longest hind toe. In addition, the distance between the posterior end of the cranium and the posterior end of the pelvis was measured as an estimate of body length. This metric was included to allow size correction of morphometric variables, including osteoderm thickness and hindlimb measurements, in subsequent comparative analyses. To account for ontogenetic variation in osteoderm expression [5], snout–vent length (SVL) was additionally measured from the micro-CT scans and expressed as the percentage of maximum SVL attained. The maximum SVL of each species was obtained from the SquamBase database [6]. In cases where the measured specimen exceeded the reported maximum SVL, the measured SVL (indicated by an asterisk in the dataset) was used as the reference maximum. This variable provides an estimate of relative size attained at the time of sampling and can be used to account for potential ontogenetic effects in subsequent analyses.

### 4.2 Climatic data

Species distribution polygons were obtained from the Global Assessment of Reptile Distributions (GARD) database version 1.1 [7]. Distribution shapefiles were imported into R and used to extract 32 environmental variables across each species’ geographic range using the R package *raster* version 3.0.7 [8]. These environmental variables included (i) 19 bioclimatic variables related to temperature and precipitation and elevation obtained from the WorldClim v. 2.1 database [9], together with solar radiation and wind speed derived from the monthly WorldClim climate layers; (ii) global cloud cover was obtained from [10]; (iii) the global aridity index obtained from the CGIAR-CSI v. 2 database (Consortium for Spatial Information) [11]; and (iv) tree cover obtained from Moderate Resolution Imaging Spectroradiometer (MODIS) data [12]. All environmental variables were obtained at a spatial resolution of approximately 1 km², except percentage tree cover which was obtained at a spatial resolution of 250 m². Solar radiation and wind speed represent annual means calculated from the corresponding monthly raster layers. Environmental values were extracted for all grid cells intersecting each species’ range polygon and averaged to obtain species-level estimates.

### 4.3 Predation risk

**Fig. 2:**
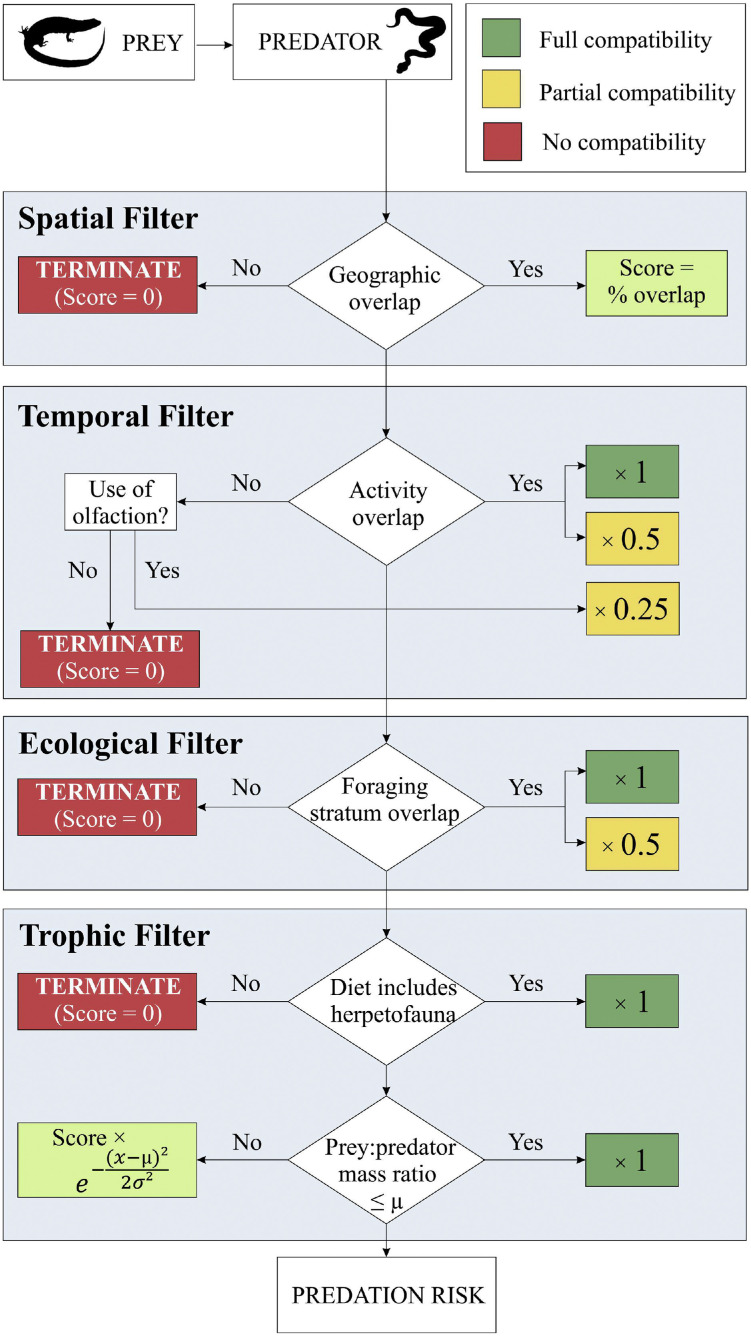
Workflow illustrating the calculation of estimated predation risk. Predation risk for each lizard species was derived using a trait-based scoring framework that integrates predator–prey spatial overlap with ecological compatibility filters. The workflow includes (1) determination of geographic range overlap between prey and predator species, (2) comparison of activity patterns, foraging strata, diet, and sensory modality, and (3) adjustment for predator–prey body size relationships using a Gaussian weighting function. Guild-specific predation risk scores were calculated for birds, mammals, and snakes, and combined to generate total and relative predation risk metrics for each prey species. Intermediate calculations for each predator guild are provided in separate Excel sheets included in the dataset.

Predation risk was estimated using a trait-based scoring framework that integrates ecological information on predator species with spatial overlap between predators and prey (Fig. 2), similar to that of [2]. A list of potential predators was compiled, including snakes (*n* = 2998 species), carnivorous mammals (*n* = 1479 species), and predatory birds (*n* = 1014 species). For each predator species, information on activity pattern, hunting strategy, diet, habitat use (foraging stratum), sensory modality (olfactory versus visually oriented predation), and body mass was compiled from published literature and trait databases, including EltonTraits 1.0 [13], PHYLACINE 1.2 [14] and SquamBase [6].

Predation risk for each predator–prey pair was calculated using a sequential filtering and weighting procedure (Fig. 2). First, geographical overlap between predator and prey distributions was calculated using species range polygons obtained from the GARD database for snakes [7], the IUCN Red List spatial database for mammals [15], and BirdLife International for birds [16]. Polygon shapefiles were loaded into R and pairwise spatial overlap between all predator and prey species was calculated using the *pairwiseRangemaps* function from the package *fuzzySim* [17]. If no spatial overlap occurred, the predation score was set to zero. If spatial overlap was present, the initial score was defined as the percentage of the prey’s geographic range that overlaps with the predator’s range.

This initial score was subsequently modified using ecological compatibility filters (Fig. 2). (1) Predator and prey activity patterns (e.g., diurnal, nocturnal, cathemeral) were compared; when activity periods overlapped, the score was retained and further adjusted according to the degree of activity match. Exact matches retained the full score, whereas partial overlaps were weighted by 0.5. (2) For predators relying primarily on olfactory cues, a lower weighting factor (0.25) was applied to account for reduced dependence on temporal activity overlap. (3) The compatibility of predator and prey foraging strata (e.g., terrestrial, arboreal, saxicolous) was evaluated. Predators occupying the same stratum as the prey retained the full score when the match was exact, whereas partial overlap resulted in a weighting factor of 0.5. If no overlap occurred, the score was set to zero. (4) Dietary information was then used to determine whether the predator is known to consume reptiles or other herpetofauna. Predators lacking herpetofauna in their documented diet received a score of zero, whereas predators known to consume reptiles retained the score. (5) Predator–prey body mass relationships were incorporated. If the prey–predator mass ratio fell within the expected predation window (i.e., less than or equal to the estimated optimal threshold μ), the score remained unchanged. For larger ratios exceeding this threshold, the score was reduced according to a Gaussian weighting function:

$$\mathrm{Score}\times e^{-\frac{{\left(x-\mu \right)}^{2}}{2\sigma^{2}}}$$

where *x* represents the predator–prey mass ratio, *μ* the estimated optimal prey size relative to predator mass, and *σ* the variance parameter describing the breadth of the prey size distribution.

The resulting value represents the estimated predation risk score imposed by a given predator species on each lizard species. Predation risk scores were subsequently summed across predator species within each predator guild (birds, mammals, and snakes) to obtain guild-specific predation risk estimates. These values were then used to calculate the relative predation risk imposed by each guild as the proportion of total predation risk contributed by that guild relative to the combined risk from all predators.

### 4.4 Life history traits

Additional life-history traits relevant to examining ecological and evolutionary correlates of armour were obtained from the global lizard trait compilation presented in [18]. These traits included activity pattern, habitat use, foraging strategy, reproductive mode, sociality and insularity. In addition, the presence or absence of a serpentiform body plan was included, as this morphology directly influences locomotor performance and may affect analyses of trade-offs between speed and armour.

### 4.5 Phylogeny

Phylogenetic relationships were derived from the comprehensive squamate phylogeny published by Tonini et al. [19]. In the original study, a molecular backbone based on sequence data for 17 mitochondrial and nuclear genes was inferred using maximum-likelihood methods implemented in RAxML and ExaML. Divergence times were estimated under a relaxed molecular clock framework with divergence-time calibrations derived from stratigraphic and molecular evidence, producing a posterior distribution of 10,000 fully sampled phylogenetic trees containing 9754 squamate species. For the present study, the time-calibrated consensus tree from this posterior distribution [19] was used as the phylogenetic framework. The tree was pruned to retain only the species included in the present dataset. Species names were subsequently updated to reflect current taxonomy following the Reptile Database [20]. Two alternative phylogenetic trees were generated: one excluding outgroups and one including two basal taxa (*Sphenodon punctatus* and *Gekko gecko*) and two additional taxa representing sister lineages to the respective focal clades (*Tiliqua scincoides* for Cordyliformes and *Iguana iguana* for Anguimorpha), to ensure appropriate rooting and comparative structure across both clades.

## Limitations

Micro-CT scans used in this dataset were obtained from multiple institutions and therefore vary in spatial resolution and scanning parameters. Although surface determination was performed manually by a single author to minimise measurement error, differences in voxel resolution may influence the precision of measurements for very small or thin osteoderms [21]. For this reason, spatial resolution metadata are included in the dataset and can be incorporated as a covariable in future analyses to account for potential resolution-related effects.

Sexual dimorphism in osteoderm expression has been reported in some lizard species [5]. Because the sex could not be determined for all specimens and sample sizes were limited for many species, the dataset does not explicitly account for sex-specific variation. When multiple specimens were available, measurements represent averages across individuals. In addition, osteoderms may develop fully only after sexual maturity [5]. Although only subadult and adult specimens were included, some ontogenetic variation among specimens may remain. To account for such effects, the dataset includes the percentage of maximum recorded SVL attained for each species.

Environmental variables were extracted from species distribution polygons rather than point occurrence records. While this approach may not capture fine-scale environmental heterogeneity within species’ ranges, it provides a standardised representation of species distributions and reduces potential sampling biases associated with uneven occurrence data availability among regions, and ensured consistency with the use of the same polygon shapefiles for calculating predator–prey spatial overlap in the predation risk framework.

Finally, phylogenetic relationships were derived from a large-scale published squamate phylogeny [19], which incorporates taxonomic placement for species lacking genetic data and therefore contains some uncertainty in the placement of poorly sampled taxa.

## Ethics Statement

The authors have read and followed the ethical requirements for publication in Data in Brief and confirm that the work did not involve human subjects, animal experiments, or any data collected from social media platforms.

## CRediT Author Statement

**Chris Broeckhoven:** Conceptualization, Methodology, Data collection, Data curation, Writing, Original draft preparation; **Bryan Minne:** Data collection; **Cang Hui:** Conceptualization, Validation, Writing- Reviewing and Editing.

## Data Availability

ZenodoArmourTraits (Original data).
